# EPA and DHA Enhance CACT Promoter Activity by GABP/NRF2

**DOI:** 10.3390/ijms25169095

**Published:** 2024-08-22

**Authors:** Eleonora Stanca, Francesco Spedicato, Anna Maria Giudetti, Laura Giannotti, Benedetta Di Chiara Stanca, Fabrizio Damiano, Luisa Siculella

**Affiliations:** 1 Department of Experimental Medicine (DiMeS), University of Salento, 73100 Lecce, Italyluisa.siculella@unisalento.it (L.S.); 2 Department of Biological and Environmental Sciences and Technologies (DiSTeBA), University of Salento, 73100 Lecce, Italyanna.giudetti@unisalento.it (A.M.G.)

**Keywords:** CACT, GABP/NRF2, n-3 PUFA, fatty acid β-oxidation

## Abstract

Carnitine-acylcarnitine translocase (CACT) is a nuclear-encoded mitochondrial carrier that catalyzes the transfer of long-chain fatty acids across the inner mitochondrial membrane for β-oxidation. In this study, we conducted a structural and functional characterization of the CACT promoter to investigate the molecular mechanism underlying the transcriptional regulation of the CACT gene by n-3 PUFA, EPA and DHA. In hepatic BRL3A cells, EPA and DHA stimulate CACT mRNA and protein expression. Deletion promoter analysis using a luciferase reporter gene assay identified a n-3 PUFA response region extending from −202 to −29 bp. This region did not contain a response element for PPARα, a well-known PUFA-responsive nuclear receptor. Instead, bioinformatic analysis revealed two highly conserved GABP responsive elements within this region. Overexpression of GABPα and GABPβ subunits, but not PPARα, increased CACT promoter activity, more remarkably upon treatment with EPA and DHA. ChIP assays showed that n3-PUFA enhanced the binding of GABPα to the −202/−29 bp sequence. Furthermore, both EPA and DHA induced nuclear accumulation of GABPα. In conclusion, our findings indicate that the upregulation of CACT by n3-PUFA in hepatic cells is independent from PPARα and could be mediated by GABP activation.

## 1. Introduction

Dietary macro and micronutrients have been recognized to finely regulate gene expression [1], modifying genome organization through epigenomic mechanisms or modulating transcription factor activity. Long-term consumption of diets rich in fats and carbohydrates can induce metabolic disorders such as dyslipidemia, steatosis [2], cardiovascular diseases, inflammation and insulin resistance [3,4,5]. These metabolic diseases are often associated with elevated levels of plasma free fatty acids and the dysregulation of fatty acid catabolism. 

Long-chain fatty acids (LCFA) serve as potent regulators of cellular metabolism, influencing the expression of genes involved in carbohydrate and lipid metabolism [1,6,7,8]. Among LCFA, polyunsaturated fatty acids (PUFA) play a pivotal role. In particular, it has been well-established that n-3 PUFA has hypocholesterolemic, hypotriglyceridemic and anti-inflammatory effects [9,10], mainly attributed to the upregulation of key enzymes involved in fatty acid oxidation [6,11] and down-regulation of several proteins involved in the lipogenic pathway [12,13,14,15]. Indeed, carnitine palmitoyl transferase 1 (CPT1), a key enzyme of mitochondrial fatty acid β-oxidation, and acyl-CoA oxidase (AOX), the first enzyme of the peroxisomal β-oxidation pathway, have been reported to be stimulated by n-3 PUFA [9,16,17]. 

Mammalian fatty acid oxidation primarily occurs in the mitochondrial matrix, where LCFA are transported via the carnitine shuttle. CPT1, located in the outer mitochondrial membrane, converts LCFA-CoA into corresponding carnitine esters, facilitating their transport across the inner mitochondrial membrane by carnitine/acylcarnitine translocase (CACT). Carnitine palmitoyl transferase 2 (CPT2) reconverts long-chain acylcarnitine into acyl-CoA esters for degradation inside the mitochondrial matrix [18].

CACT is a nuclear-encoded carrier protein embedded in the inner mitochondrial membrane and is encoded by the *Slc25a20* (also named *Cact*) gene belonging to the SLC25 gene family. CACT plays a prominent role in the mitochondrial β-oxidation of LCFA resulting from both dietary sources and adipose tissue lipolysis. Mutations in the human *Cact* gene cause a rare but severe fatty acid oxidation disorder known as CACT deficiency [19]. Clinical manifestations of the disease, common to other β-oxidation defects, include neurological dysfunctions, cardiomyopathy, skeletal muscle damage and liver impairment [20]. Treatments for CACT deficiency involve prevention of fasting, administration of high carbohydrate and low LCFA diets or PUFA- and medium-chain triglyceride-based supplements [21]. 

Dietary fatty acid composition has been shown to influence the expression and activity of CACT in rat liver mitochondria. Compared to diets enriched in saturated fatty acids, those enriched in fish oil (FO) have been reported to increase CACT expression, while diets enriched in olive oil (OO) have a slight inhibitory effect [22]. Eicosapentaenoic acid (EPA; 20:5n-3) and docosahexaenoic acid (DHA; 22:6n-3) are the most relevant n-3 PUFA components of FO involved in *Cact* gene regulation [23,24].

This study aims to investigate the molecular mechanism underlying the transcriptional regulation of the *Cact* gene by EPA and DHA through the structural and functional characterization of its promoter region.

## 2. Results

### 2.1. Fatty Acids Differently Affect CACT Expression

**Figure 1:**
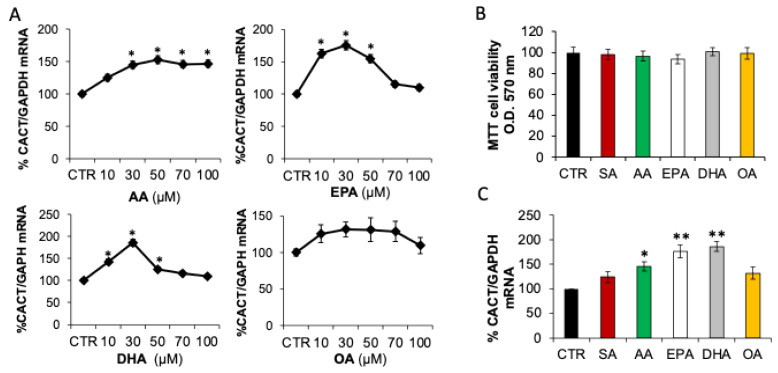
Unsaturated fatty acids increased CACT mRNA levels. (**A**) Hepatic cells were incubated with BSA (control cells, CTR) or indicated concentration of AA, EPA, DHA and OA for 24 h and then RNA was extracted. CACT mRNA was quantified by Real-time PCR, and *Gapdh* was used as a housekeeping gene for normalization. (**B**) Proliferation and viability analysis of BRL3A treated with 30 μM LCFA or BSA (CTR) for 24 h by MTT assay. (**C**) Hepatic cells were incubated with 30 μM LCFA or BSA (CTR) for 24 h and then RNA was extracted. CACT mRNA was quantified by Real-time PCR, and *Gapdh* was used as a housekeeping gene for normalization. Results are expressed as the mean ± SD of triplicate measurements from three independent experiments. * *p* < 0.05, ** *p* < 0.01 versus control.

In a previous study, it has been reported that saturated and unsaturated fatty acid-enriched diets differently modulated the expression and the activity of CACT in rat liver mitochondria [22]. In order to investigate the mechanism underlying the transcriptional regulation of the *Cact* gene, the effect of exogenous fatty acids on CACT expression was investigated in hepatic BRL3A cells. Hepatic cells were treated with arachidonic acid (C_20:4,n-6_, AA), EPA, DHA and oleic acid (C_18:1_, OA) at concentrations ranging from 10 to 100 µM (Figure 1A). MTT testing showed that the treatments did not affect the cell viability (Figure 1B). We observed that treatments with PUFA increased the CACT mRNA abundance, although the dose-dependent profile of the mRNA changes was different depending on the type of PUFA. Treatment with AA caused a dose-dependent increase in CACT mRNA levels, with a maximum of about 45% in cells incubated with 50 µM of fatty acids with respect to control cells. Treatment with 30 µM EPA or DHA resulted in a maximum increase in CACT mRNA of about 76% and 96%, respectively, compared to untreated cells. Conversely, the increase in CACT mRNA abundance was attenuated or reversed at 50–100 µM of PUFA (Figure 1A).

Our findings suggested that alterations in CACT mRNA levels were particularly induced by n-3 PUFA, reaching the maximum stimulation at the concentration of 30 µM. No significant increment was observed after OA treatment as compared to both stearic acid (C_18:0_, SA) and the control (Figure 1C). 

### 2.2. Structural Organization of CACT Promoter and 5′-UTR 

The increase in CACT mRNA abundance observed in PUFA-treated hepatocytes could be associated with the transcriptional regulation of the gene. For this purpose, the CACT promoter has been characterized through the identification of the transcription start site.

**Figure 2:**
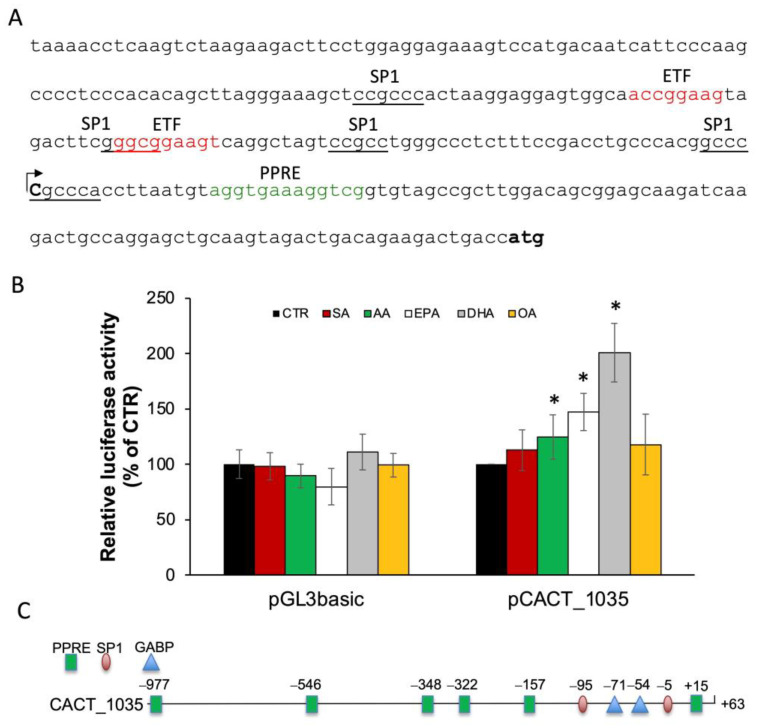
Analysis of proximal promoter region and 5′UTR of rat *Cact* gene. (**A**) The sequence of the 291 nucleotides upstream of the translation start codon of the rat *Cact* gene is shown; the transcription start site and putative responsive elements Sp1 (underlined), ETF (red) and PPRE (green) are indicated. (**B**) Effect of 30 mM LCFA or BSA (CTR) on the pCACT_1035 promoter activity in BRL3A cells. Results are expressed as the mean ± SD of triplicate measurements from three independent experiments. * *p* < 0.05 (**C**) Schematic diagram of pCACT_1035 showing transcription factor binding sites for SP1, GABP and PPARα.

To define the transcription start site, 5′ RACE was carried out, and only one amplimer was obtained, which was subjected to sequence analysis. The alignment of the obtained sequence with the *Cact* gene present in the database (accession number NW_047802) was used to identify the transcription start site represented by a cytidine located 100 bp upstream of the ATG codon (Figure 2A). Once the transcription start site was defined, a luciferase reporter construct was obtained by cloning a proximal promoter region of 1098 bp extending from −1035 to +63 into pGL3-basic, obtaining the pCACT_1035. Upon transfection with the pCACT_1035, cells were treated with 30 µM of saturated (SA) or unsaturated (OA, AA, EPA, DHA) fatty acids for 24 h. Transfection with the empty pGL3-basic was performed as a negative control. Results showed that EPA and DHA increased CATC promoter activity by about 47% and 107%, respectively, compared to the untreated cells (Figure 2B).

To identify DNA cis-regulatory elements responsible for transcriptional regulation of CACT by PUFA, bioinformatics analysis of the *Cact* gene promoter was performed. Promoter analysis showed four SP1 binding sites at −93 bp, −54 bp, −35 bp and −4 bp, five putative PPAR response element (PPRE) sites at positions −966 bp, −546 bp, −341 bp, −322 bp and +15 bp and also GABP/NRF2 consensus sequences at −70 bp and −53 bp (Figure 2C). The sequence of the binding sites for each transcription factor showed a core identity greater than 90% with respect to the corresponding consensus sequence.

### 2.3. n-3 PUFA Increased CACT Promoter Activity

**Figure 3:**
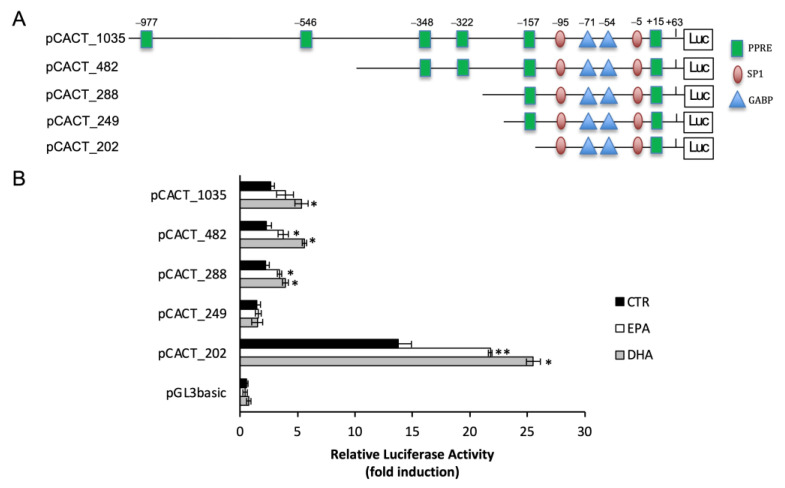
EPA and DHA increased CACT promoter activity. (**A**) Constructs with 5′ deletion end point used for transient transfection to identify transcription factor binding site. (**B**) Effect of n-3 PUFA on the promoter activity of CACT in BRL3A cells. Deletion fragments of the promoter were cloned into pGL3-basic vector upstream of the LUC gene coding sequence. Relative luciferase activity is shown as the ratio of Firefly/Renilla luciferase and bars represent means ± SD of at least three independent experiments. * *p* < 0.05, ** *p* < 0.01 versus control.

To identify the DNA sequence responsible for the n-3 PUFA effect on CACT mRNA expression, a series of truncated regions of the CACT promoter were cloned into a pGL3_basic luciferase reporter vector. These constructs, reported in Figure 3A, were used to transiently transfect BRL3A cells. To rule out the non-specific effect of PUFA on the expression of the reporter gene, transfection with the empty plasmid pGL3-basic was carried out as a control. After 24 h treatment with EPA and DHA, cells were lysed and assayed for luciferase activity. When compared to the full-length promoter (pCACT_1035), serial deletion constructs showed similar responsiveness to n-3 PUFA treatments (Figure 3B). The luciferase activity in cells transfected with pCACT_482, pCACT_288 and pCACT_202 increased by about 55% and 90% after treatment with EPA and DHA, respectively, compared to untreated cells. The deletion construct pCACT_249 showed a reduced basal activity by about 80% as compared to pCACT_1035 basal activity, while n-3 PUFA effect on transcriptional activity was abolished. However, the uninduced basal activity of the shorter construct, pCACT_202, increased by 4.1-fold as compared to CACT_1035 basal activity, and the induction of luciferase activity by EPA and DHA treatment was restored. These data suggested that PUFA-responsive elements may be located between −202 and +63 of the *Cact* gene in hepatic cells. 

It is known that n-3 PUFA can activate PPAR nuclear receptors [6]. The pCACT_202 construct has a putative PPRE site in the 5′-UTR at +15 position, which shows high similarity to the PPRE consensus motif [25]. 

**Figure 4:**
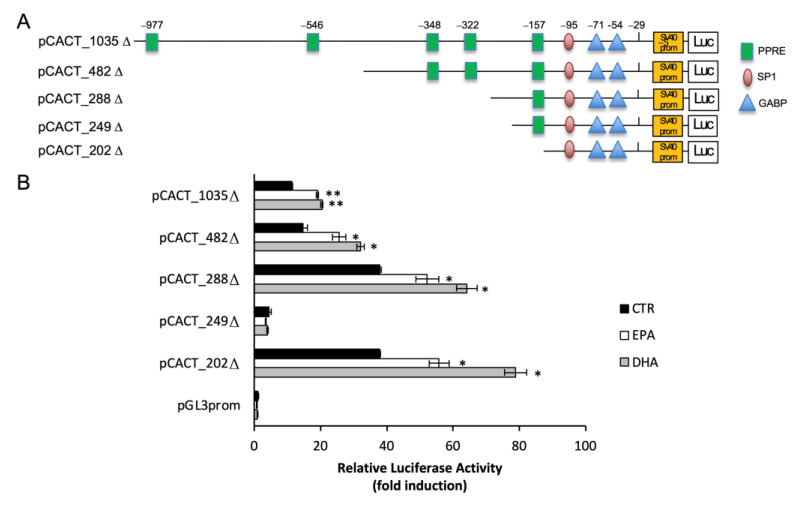
The effect of EPA and DHA on CACT promoter activity deleted of 5′UTR elements. (**A**) CACT promoter region with 5′ deletion endpoint and deletion of 28 nucleotides upstream +1 nucleotide. (**B**) Effect of n-3 PUFA on the promoter activity of CACT in BRL3A cells. Deletion fragments of the promoter were cloned into the pGL3_promoter vector upstream of the LUC gene coding sequence. Relative luciferase activity is shown as the ratio of Firefly/Renilla luciferase and bars represent means ± SD of at least three independent experiments. * *p* < 0.05, ** *p* < 0.01 versus control.

To assess whether the PPRE element +15 was responsible for PUFA responsiveness of the *Cact* gene, promoter deletion fragments were obtained by PCR without the sequence from −29 bp to +63 bp. The resulting amplimers were cloned into pGL3_promoter luciferase reporter vector (Figure 4A) and BRL3A cells were transiently transfected with the five deletion promoter constructs lacking the +15 PPRE: pCACT_1035Δ, pCACT_482Δ, pCACT_288Δ, pCACT_249Δ and pCACT_202Δ. After treatment with EPA and DHA, cells were lysed and assayed for luciferase activity. Treatment with EPA and DHA induced luciferase activity of the promoter-reported constructs by about 56% and 94%, respectively, compared to the control (Figure 4B). These data suggest that PPARα may not be involved in the regulation of CACT expression mediated by n-3 PUFA. Moreover, PUFA-responsive elements were located between −202 and −29 bp of the CACT promoter.

### 2.4. Effect of the PPARα Agonist WY-14,643 on CACT Expression and Promoter Activity

**Figure 5:**
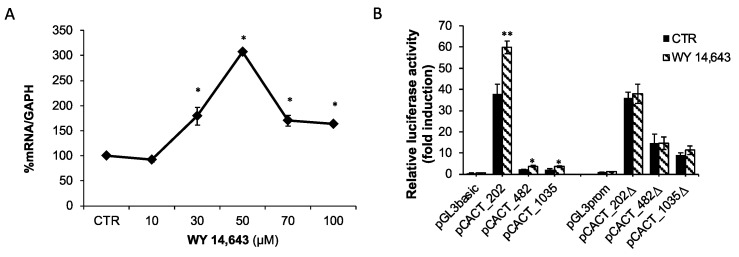
PPARα induced CACT expression through PPRE element at +15. (**A**) CACT mRNA levels were quantified in BRL3A cells treated with increasing concentrations of PPARα agonist. (**B**) Effect of PPARα agonist on the promoter activity of CACT in BRL3A cells. Deletion fragments of promoter were cloned into pGL3-basic or pGL3-promoter vector upstream of the LUC gene coding sequence. Relative luciferase activity is shown as the ratio of Firefly/Renilla luciferase and bars represent means ± SD of at least three independent experiments. * *p* < 0.05, ** *p* < 0.01 versus control.

To elucidate the role of PPARα in the regulation of CACT expression by EPA and DHA, hepatic BRL3A cells were treated with the synthetic PPARα agonist WY-14,643. The cells were treated with increasing concentrations (from 10 μM up to 100 μM) of the synthetic PPARα agonist, for 24 h. CACT mRNA was increased by about 79%, 207% and 63% in the presence of 30 μM, 50 μM and 100 μM WY-14,643, respectively (Figure 5A). To demonstrate that the CACT promoter is activated by the PPARα agonist, BRL3A cells were transfected with the promoter-reporter constructs described above, in the presence or absence of the PPRE +15 element, and then incubated with 30 μM WY-14,643 for 24 h. The transcriptional activity of pCACT_1035, pCACT_482 and pCACT_202 was induced by about 60% after WY-14,643 treatment (Figure 5B). Otherwise, the constructs pCACT_1035Δ, pCACT_482Δ and pCACT_202Δ showed no induction of promoter activity after WY-14,643 treatment (Figure 5B), indicating that the PPRE at position +15 is a PPARα binding and activation site. 

### 2.5. n-3 PUFA Enhanced CACT Expression via GABP/NRF2

**Figure 6:**
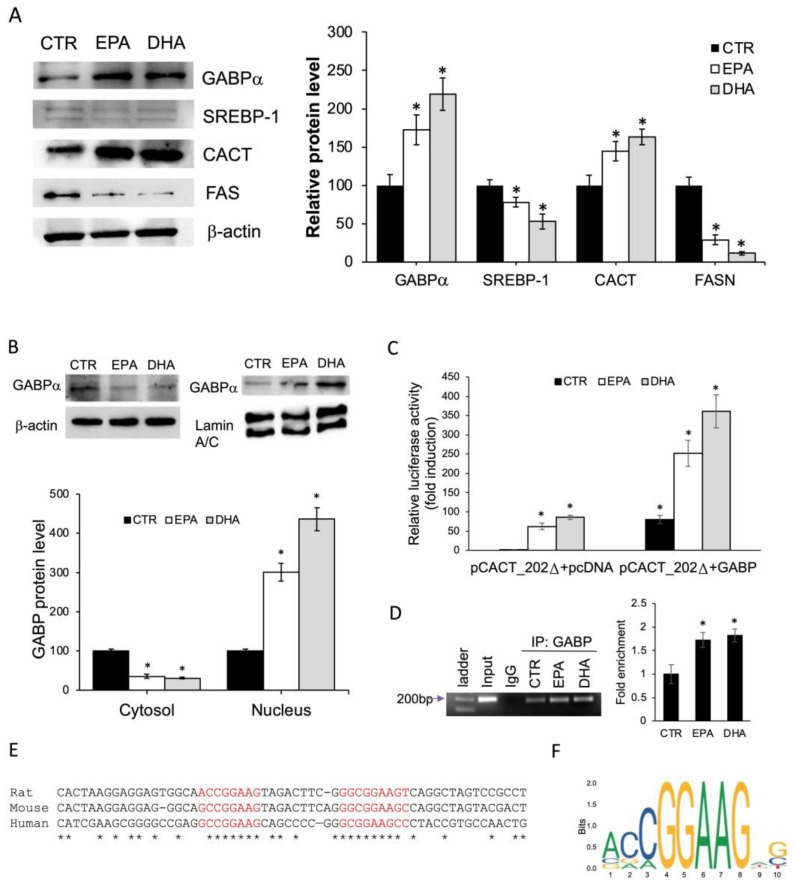
n-3 PUFA induced CACT expression through GABP binding to the CACT promoter. (**A**) Expression protein levels in total protein extracts of BRL3A cells incubated with 30 μM EPA, DHA or BSA(CTR) for 24 h. Western blots were probed with specific primary antibodies against SREBP-1, FAS, GABPα and CACT. (**B**) GABPα protein levels in cytosolic and nuclear protein extracts of BRL3A cells treated with 30 μM EPA, DHA or BSA (CTR). Blots of cytosolic and total extracts were normalized vs. b-actin and blots of nuclear extracts vs. lamin A/C. The results were expressed as the mean ± SD of triplicate measurements from three independent experiments. * *p* < 0.05 versus control. (**C**) BRL3A cells were transiently co-transfected with pCACT_202Δ and GABP expression plasmid. Subsequently, cells were treated with 30 μM EPA, DHA or BSA (CTR) for 24 h. Afterward, cells were lysed, and luciferase activities were determined. Relative luciferase activity is shown as the ratio of Firefly/Renilla luciferase and bars represent means ± SD of at least three independent experiments. * *p* < 0.05 versus control (**D**) ChIP analysis of the CACT promoter was conducted using BRL3A cells treated with 30 μM EPA, DHA or BSA (CTR). Chromatin fragments immunoprecipitated (IP) with anti-GABP antibodies were amplified by Real-time PCR with primers spanning the two ETF motifs of the CACT promoter. Samples incubated with non-specific preimmunize IgG were used as negative controls, whereas chromatin fragments non-immunoprecipitated were used as positive controls (input). The results shown are representative of four individual experiments. Histogram showing the quantification of Real-time PCR obtained on GABPα immunoprecipitated DNA, relative to input signal; the relative enrichment of CACT DNA of CTR was fixed as 1. The results were expressed as the mean ± SD of measurements from four independent experiments. * *p* < 0.05 versus control. (**E**) Alignments of rat, mouse and human CACT promoter regions containing the two putative GABP binding sites are shown. Matching nucleotides are shown by asterisks. (**F**) MEME logo of the GABP motif of the human consensus sequence was obtained using open-access database Jaspar.

Bioinformatic promoter analysis identified two GABP/NRF2 responsive elements at −54 bp and at −71 bp, both located in the shorter promoter-reporter construct, whose activity was induced by n-3 PUFA. First, we investigated the effect of n-3 PUFA treatment on GABP protein levels. Western blot analysis showed that EPA and DHA caused an increase in GABP protein levels by about 73% and 119%, respectively, compared to the control. EPA and DHA treatments upregulated CACT protein levels, with respect to the control, by about 48% and 63%, respectively. As expected, we found a marked reduction of SREBP-1 and FASN protein levels by EPA and DHA (Figure 6A). Moreover, subcellular fractionation analyses revealed that n-3 PUFA induced nuclear GABP translocation, with decreased cytoplasmic GABP protein levels compared to the control. Conversely, an increase of GABP protein levels in nuclear fraction by 201.7% and 336.4% of BRL3A treated with EPA and DHA, respectively, was observed (Figure 6B).

To investigate the ability of GABP to mediate the CACT promoter response to n-3 PUFA, co-transfection of rat GABPα and GABPβ expression plasmid [26] together with the promoter-reporter construct pCACT_202Δ was performed. Compared to the control, overexpression of GABPα and GABPβ increased the luciferase activity by about 3.5- and 2.5-fold in cells treated with DHA and EPA, respectively; this increase was significantly higher than the luciferase activity recorded in the absence of exogenous expression of the transcriptional factor (Figure 6C). 

Furthermore, to determine the binding of GABP to the proximal promoter of the endogenous *Cact* gene and its involvement in n-3 PUFA-induced CACT regulation, Chip assay was performed. Chromatin from BRL3A cells incubated for 24 h in DMEM supplemented with BSA (control), DHA or EPA was isolated. After immunoprecipitation with antibodies against GABPα or with non-specific IgGs, Real-time PCR was performed using specific primers to amplify the proximal CACT promoter region at −202 to −29. The presence of the 197 bp amplicon revealed that the transcription factor GABP bind to the CACT promoter in vivo. Treatment of the BRL3A cells with EPA and DHA increased the binding of GABP to the proximal promoter (Figure 6D). 

Sequence alignment of the human, rat and mouse CACT promoters revealed that the two GABP responsive elements shared high sequence homology (Figure 6E) and the GABP responsive element at −71 bp was similar, although slightly shorter (two nucleotides) than the canonical consensus sequence found in the Jaspar open-access database (Figure 6F). 

## 3. Discussion

In this study, the proximal CACT promoter region was structurally and functionally characterized for its responsiveness to n-3 PUFA. For the first time, two GABP/NRF2 responsive elements located in the 5′ flanking regulatory region of the *Cact* gene were identified and their involvement in the upregulation of CACT expression induced by n-3 PUFA was demonstrated. 

n-3 PUFA, such as EPA and DHA, play an important role in the regulation of lipid metabolism. Dietary n-3 PUFA supplementation reduces triglyceridemia, white adipose tissue and hepatic steatosis [27,28]. These effects are due to the activation of anti-inflammatory pathways [29] and the regulation of the expression of genes involved in fatty acid absorption, transport, oxidation and synthesis [6,28]. n-3 PUFA play an important role in upregulating fatty acid oxidation, and their administration increases the expression of CPT1 and AOX [30,31] as well as transcription factors like PPARα that modulate the expression of genes involved in fatty acid oxidation [6]. 

In this work, we observed that treatment with PUFA, and in particular n-3 PUFA, increased the mRNA levels of CACT in BRL3A hepatic cells compared to control cells and those treated with SA and OA. These data fit perfectly with the increase in CACT expression and activity observed after a FO- and krill oil-supplemented diet administration to rodents [22,28]. The main fatty acid components contained in these oils are EPA and DHA. Furthermore, our data is consistent with the increase of CPT1 gene expression induced by EPA and DHA treatment [32,33].

CACT promoter analysis showed the absence of TATA box sequence; however, we identified Sp1 binding sites, near the transcriptional start site, that are required to initiate transcription [34], as occurs in other metabolic genes [13]. CACT is predominantly expressed in tissues where β-oxidation is active, such as the kidney, muscle, heart, brain and notably the liver [35]. Mutations in the gene cause disorders in mitochondrial long-chain fatty acid β-oxidation, with variable clinical symptoms: cardiac arrhythmia, lethargy, hypoglycemia, hypotonia, liver dysfunction and brain injury. Therapies used to limit side effects of diseases cause a deficiency of PUFA in the plasma, which is compensated for by dietary DHA supplementation [36]. 

The CACT promoter has been well characterized [25,37,38,39,40]; however, the effect of n-3 PUFA on the CACT promoter has not been studied to date. The *Cact* gene promoter exhibits a functional conserved PPRE [25,37,38]. It is well known that n-3 PUFA are important positive regulators of PPARα. Bioinformatic analysis of the proximal CACT promoter region identified five putative PPRE. n-3 PUFA induced a significant increase in CACT promoter activity, consistent with up-regulation of CACT mRNA and protein levels in n-3 PUFA-treated BRL3A cells, as previously observed in the liver of rats fed on a FO-supplemented diet [22]. 

Unexpectedly, an assessment of CACT promoter activity showed that the removal of all PPRE elements did not reduce luciferase expression after n-3 PUFA treatment. Otherwise stated, the smaller promoter fragment extending from +202 to +29 contains cis-acting elements responsible for PUFA-induced CACT activity. These data suggest that PUFA can activate CACT transcription via a pathway independent of PPARα. A similar regulatory mechanism has been reported for LCFA-induced CPT1 gene transcription in rat hepatocytes whereas the muscle isoform is upregulated by LCFA through PPARα binding to PPRE consensus sequences [41]. In the deletion promoter analysis, we observed that pCACT_249 had low promoter activity and was unresponsive to PUFA. Conversely, all the other constructs, including the shorter pCACT_202, exhibited responsiveness to PUFA. This discrepancy can be explained by the presence of binding sites for negative regulators in the fragment extending from +249 to +202, whose activity could be modulated depending on the promoter context. Further experiments are required to elucidate the regulation of the CACT promoter by putative negative regulators. 

To confirm that the increase in CACT promoter activity in the absence of PPRE-responsive elements was specific to fatty acids, CACT expression and its promoter activity were assayed in the presence of the PPARα agonist. WY-14,643 was able to induce an increment of CACT mRNA and of CACT promoter activity; notably, in the absence of PPRE at position +15, WY-14,643 failed to induce luciferase activity. The PPARα responsive element located at position +15 relative to the transcription start site is conserved in humans and it is responsible for inducing CACT expression in response to statins, fibrates and PPARα and PPARδ agonists [25,37]. 

Here, we have identified, in the smaller promoter region of CACT, two adjacent binding sites for the transcription factor GABP. Many nuclear-encoded mitochondrial proteins have GABP-responsive elements in their promoters. Specifically, two proteins involved in the termination and initiation steps of transcription, mTERF (mitochondrial termination factor) and POLRMT (mitochondrial termination factor), show two tandem GABP responsive elements in the proximal promoters of their genes [42]. GABP, along with other transcription factors such as NRF1, Sp1, PGC1α and ERRα, is involved in the regulation of mitochondrial biogenesis and oxidative phosphorylation [43,44]. GABP, also named NRF2, belongs to the ETS (E26 transformation-specific) transcription factor family. It is an obligate tetramer consisting of two GABPα and two GABPβ subunits that form the transcriptionally active complex; GABPα contains the ETS DNA binding domain and can recruit GABPβ [44].

To demonstrate GABP activation by n-3 PUFA, we investigated the GABPα protein levels in total lysate, cytosolic and nuclear fractions. Treatment with EPA, and especially with DHA, strongly increased the expression of GABPα. Conversely, a significant decrease in the expression of SREBP1 and FAS, two key regulators of lipogenesis, was observed. Interestingly, treatment with n-3 PUFA increased GABPα protein levels in the nuclear fraction compared to the control. This suggests that n-3 PUFA not only increased the expression of GABPα but also induced nuclear translocation of the transcription factor. 

The magnitude of GABP upregulation exceeded the increase in CACT, which could correlate with other functions played by GABP that may be linked to PUFA treatment. Recently, the involvement of GABP in reducing lipotoxicity and inflammation in myosteatosis has been demonstrated [45]; a similar effect was observed after n-3 PUFA treatment [46,47]. Conversely, an increase in reactive oxygen species in muscle cells (C2C12) mediated the dissociation of the active GABP tetramer complex [48]. Nevertheless, GABP, regulated by RORa, is involved in enhancing fatty acid oxidation in a myosteatosis model [45]. These data are consistent with ours, albeit in a different experimental model. However, to date, there is no evidence correlating the action of n-3 PUFA with the GABP transcription factor. 

Our data indicate that overexpression of GABPα and GABPβ in BRL3A cells resulted in a significant increase in CACT promoter transcriptional activity both in the presence or absence of EPA and DHA. Furthermore, ChIP analysis revealed that the CACT promoter fragment of 172 bp containing ETS-responsive elements co-precipitated with anti-GABPα antibodies. The specific amplicon disappeared in chromatin immunoprecipitates obtained with nonspecific IgG, indicating that the transcription factor was associated with the CACT proximal promoter in vivo. The amount of amplimer detected in immunoprecipitates from cells treated with EPA or DHA was greater than in the control, supporting that the GABP transcription factor is activated by n-3 PUFA.

Notably, the alignment of the CACT proximal promoter of human, rat and mouse genes showed that the two consensus sequences for GABP are extremely conserved across species. In particular, the cis-element at position −72 bp aligns with GABP/NRF-2 binding sites located in the promoters of mitochondrial biogenesis genes [42] and with the ETS-related factors motif reported in the Jaspar database. The presence of a GABP-responsive functional element in the CACT promoter is confirmed by ChIP-seq analysis in the INS-1 cell. In this model, it has been demonstrated that GABP siRNA knockout reduced CACT mRNA levels [49]. Further experiments are needed to explain how n-3 PUFA influences the action of the GABP transcription factor. 

In conclusion, our findings indicate that: (i) the upregulation of CACT by n-3 PUFA treatment in the hepatic cells is independent of PPARα and (ii) CACT is regulated by GABP at the transcriptional level through the binding to GABP responsive elements located at −54 and −71 in the CACT proximal promoter.

## 4. Materials and Methods

### 4.1. Cell Culture and Treatments

BRL3A cells were maintained in William’s medium (Sigma-Aldrich, MI, Italy) supplemented with 10% (*v*/*v*) fetal bovine serum (FBS), penicillin G (100 units/mL) and streptomycin (100 µg/mL) at 37 °C under 5% CO_2_ atmosphere. Incubation with fatty acids was performed by treating BRL3A cells with 10% (*v*/*v*) delipidated FBS and containing either fatty acid-free BSA (Control, CTR) or one of the following albumin-bound fatty acids: SA, OA, AA, EPA or DHA for 24 h. The molar ratio of fatty acids and albumin was 4:1. Dose-dependent experiments were carried out incubating BRL3A cells with increasing fatty acid concentrations (10–100 μM) for 24 h. BSA and fatty acids were purchased from Sigma-Aldrich, MI.

### 4.2. Isolation of RNA and Real-Time PCR Analysis

Total RNA was extracted from BRL3A seeded in 6-well cultured plates at a density of 2 × 10^5^ for 24 h. Only RNA without DNA contamination was used for subsequent preparation of cDNA synthesis. The RT reaction (20 μL) was carried out using 1 μg of total RNA, 25 ng of random hexamers and 50 units of SuperScriptTM III RNase H-RT (Invitrogen, Monza, Italy). Quantitative gene expression analysis was performed in a CFX Connect Real-time System (BioRad, Segrate, Italy) using SYBR Green technology (FluoCycle, Euroclone, Pero, Italy). Glyceraldehyde 3-phosphate dehydrogenase (GAPDH) was used as an internal control for normalization [22] The sequences of primers used in Real-time PCR are reported in Table 1.

### 4.3. 5′ Rapid Amplification of cDNA Ends (RACE)

5′ RACE was performed using RNA isolated from rat liver. The specific cDNA was obtained with reverse primer CRT1 AGAGTCTTCCGGAAACAGTC. cDNA was ligated to the kit adaptor and amplified by PCR experiments using sets of primers complementary to the adaptor sequence and to the 5′ end of the CACT cDNA. The primers for nested PCR were CR1 TGGTGGTTGTGTCTGCAGTC and CR2 AGATCGAATTCGGGAGGTTCTTAAGCGGAC complementary to sequence within the first exon. RACE products were subcloned into pBluescript II K/S (Invitrogen, Monza) and sequenced with primers specified by the manufacturer.

### 4.4. Bioinformatics Analysis

The identification of the transcription start site was obtained through sequence alignment carried out using the BLAST program available on the site http://blast.ncbi.nlm.nih.gov/Blast.cgi (accessed on 19 June 2019). Transcription factor binding sites in the CACT promoter region were predicted using the Match and *p*-match programs at http://www.gene-regulation.com based on the TRANSFAC database. Multiple sequence alignment of the CACT promoter of different species was performed with ClustalW (http://www.ebi.ac.uk/clustalw (accessed on 25 July 2024)) Determination of GABP common motifs and graphical logo was obtained using the Jaspar database on matrix MA0062.1 (https://jaspar.elixir.no).

### 4.5. Plasmid Vector Constructs and Transient Transfection Assay 

Five DNA fragments of the rat CACT promoter, namely, pCACT_1035, pCACT_482, pCACT_288, pCACT_249 and pCACT_202, were obtained by PCR. The amplicons were digested by KpnI and HindIII and then cloned into corresponding sites of the pGL3_basic vector upstream of the LUC gene coding sequence. The primer sequences are reported in Table 1.

To study the responsiveness to fatty acids of the CACT promoter without conserved PPRE in 5′UTR, amplimers with the same forward primers and the inner −29 nt promREV1 reverse primer were obtained by PCR and cloned into the KpnI and XhoI sites of the pGL3_promoter downstream of the SV40 promoter. All the constructs were sequenced to confirm the accuracy.

After 24 h of plating, cells were co-transfected with one of the CACT promoter constructs in pGL3-basic or in in pGL3-promoter (1.5 µg/well) and the pGL4.73 Renilla luciferase reference plasmid (0.02 µg/well), a control for transfection efficiency. Transfections were carried out using FuGENE 6 transfection reagent (Roche Diagnostics, Monza, Italy), following the instructions. After a seven-hour transfection period, the medium was changed with fresh DMEM supplemented with 10% (*v*/*v*) of delipidated FBS [13] and containing either fatty acids/BSA complexes or fatty acid-free BSA as a control. Cells were incubated for 24 h, then lysed, and Firefly and Renilla luciferase activities were measured using Dual-Luciferase Reporter Assay System (Promega, Milan, Italy). 

Mammalian vectors for the overexpression of GABPα and GABPβ have been previously described [26].

### 4.6. Preparation of Nuclear and Cytosolic Protein Extracts

BRL3A cells from 100 mm plates treated with n-3 PUFA or BSA only (CTR) were pooled and centrifuged at 900× *g* for 5 min at 4 °C. The resulting cell pellet was resuspended and homogenized with a Dounce homogenizer in Buffer 1 (20 mM Tris–HCl [pH 8.0], 420 mM NaCl, 2 mM EDTA, 2 mM Na_3_VO_4_, 0.2% [*v*/*v*] Nonidet P-40, 10% [*v*/*v*] glycerol). To prevent proteolysis, a mixture of protease inhibitors from Sigma Chemical Co. (Milan, Italy) was included in all the buffers. The homogenate was centrifuged at 1100× *g* for 10 min, and the supernatant was collected as the cytosolic fraction. The nuclear pellet was washed once in Buffer 1 and then resuspended in high-salt Buffer 2 (20 mM Tris–HCl [pH 7.9], 420 mM NaCl, 10 mM KCl, 0.1 mM Na_3_VO_4_, 1 mM EDTA, 1 mM EGTA, 20% [*v*/*v*] glycerol). This suspension was incubated on ice for 30 min and then centrifuged at 15,000× *g* for 15 min. The resulting supernatant is designated as the nuclear extract fraction. The purity of the fraction was tested by immunoblotting with an anti-Lamin A/C polyclonal antibody. Protein concentration was determined using the Bio-Rad protein assay kit (Milan, Italy). Lyophilized bovine serum albumin (BSA) was used as a standard.

### 4.7. Western Blotting Analysis

An equal amount of proteins from BRL3A treated with BSA alone or fatty acids/BSA was loaded onto a 15% polyacrylamide gel and Western blot was carried out as reported in [22]. Separated proteins were then transferred electrophoretically onto a nitrocellulose membrane (Pall, East Hills, NY, USA). Equal protein loading was confirmed by Ponceau S staining. The filter was blocked with 5% (*w*/*v*) non-fat dried milk in buffered saline. Blots were incubated with primary antibodies directed against CACT (rabbit polyclonal anti-SLC25A20 from Sigma-Aldrich, MI, Italy), GABPα, SREBP1, FASN or b-actin (Santa Cruz, CA, USA), with the latter used as a control, all diluted in 20 mM Tris/HCl, pH 7.6, 0.14 M NaCl, 0.5% Tween 20 (TBS-T). After washing with TBS-T, the membranes were incubated with HRP-conjugated secondary antibodies (Amersham International, Corston Bath, UK), which were revealed using the chemiluminescence kit (Amersham International, Corston Bath, UK). Densitometric analysis was carried out on the Western blots using the ChemiDoc MP Image System (BioRad, Segrate, MI, Italy).

### 4.8. Chromatin Immunoprecipitation (ChIP) Assay 

ChIP assays were performed as described in [13]. Briefly, BRL3A (100 mm dishes) were cross-linked, scraped and lysed. The chromatin was sheared using sonication yielding fragments about 500 bp in size. Specific protein-DNA complexes were immunoprecipitated for 12–18 h at 4 °C with 10 μg of GABPα antibody or non-specific IgG on a rotating wheel. After immunoprecipitation, cross-linking was reversed, the proteins were removed by treatment with proteinase K and the DNA was extracted and used as a template in Real-time PCR. The primers used for the amplification of the CACT promoter were the following: 5′-TATGCATCTTTTCACTTTGTC-3′ and 5′-AGGCGGACTAGCCTGACTTC-3′. PCR products, corresponding to a 177 bp fragment, were separated on a 1.5% (*w*/*v*) agarose gel and analysed by ethidium bromide staining. The stained gel was visualized, and the PCR products from three individual experiments were quantified using the ChemiDoc MP Image System (BioRad).

### 4.9. Statistical Analysis 

Data are the means ± SD of the indicated number of experiments. Comparison was made using one-way ANOVA followed by an unpaired Student’s *t*-test to determine any significant differences between two groups. All statistical analyses were performed using an SPSS/PC program 23.0.0 (SPSS, Chicago, IL, USA). Differences were considered statistically significant at *p* < 0.05.

## Figures and Tables

**Table 1:** Primers used for Real-time PCR analysis and for cloning of the promoter regions.

Gene	Accession Number	Sequences 5′-3′
*Slc25a20*	NW_047802	promFOR1: GAATTCGGTACCAAATTCCTCCACGGTACCTC
		promFOR2: GAATTCGGTACCCCCAAAGAGGCCAAAAGAGG
		promFOR3: GAATTCGGTACCTTCACCTCTTTACCATGCCC
		promFOR4: GAATTCGGTACCCTATGCATCTTTTCACTTTGTC
		promFOR5: AATTCGGTACCCCACAGAGGACCAGAGAAAG
		promREV: CTCGAGAAGCTTGTCTTGATCTTGCTCCGCT
		promREV1: GGTACCCTCGAGAGGCGGACTAGCCTGACTTC
	NM_053965	FOR: GTGTGCTTCTTTGGGTTTGG REV: TTCTCCAGGGGTCATGATTC
*Gapdh*	NM_017008	REV: GCCGCCTGCTTCACCACCTTCT FOR: GCATGGCCTTCCGTGTTCCTACC

## Data Availability

Data is contained within the article.

## References

[1] Pégorier J.P., Le May C., Girard J. (2004). Control of gene expression by fatty acids. J. Nutr..

[2] Wang Y., Huang F. (2015). N-3 Polyunsaturated Fatty Acids and Inflammation in Obesity: Local Effect and Systemic Benefit. Biomed. Res. Int..

[3] van Dijk S.J., Feskens E.J., Bos M.B., Hoelen D.W., Heijligenberg R., Bromhaar M.G., de Groot L.C., de Vries J.H., Müller M., Afman L.A. (2009). A saturated fatty acid-rich diet induces an obesity-linked proinflammatory gene expression profile in adipose tissue of subjects at risk of metabolic syndrome. Am. J. Clin. Nutr..

[4] Joffe Y.T., Collins M., Goedecke J.H. (2013). The relationship between dietary fatty acids and inflammatory genes on the obese phenotype and serum lipids. Nutrients.

[5] Enos R.T., Davis J.M., Velázquez K.T., McClellan J.L., Day S.D., Carnevale K.A., Murphy E.A. (2013). Influence of dietary saturated fat content on adiposity, macrophage behavior, inflammation, and metabolism: Composition matters. J. Lipid. Res..

[6] Jump D.B., Tripathy S., Depner C.M. (2013). Fatty acid-regulated transcription factors in the liver. Annu. Rev. Nutr..

[7] Giudetti A.M., Stanca E., Siculella L., Gnoni G.V., Damiano F. (2016). Nutritional and Hormonal Regulation of Citrate and Carnitine/Acylcarnitine Transporters: Two Mitochondrial Carriers Involved in Fatty Acid Metabolism. Int. J. Mol. Sci..

[8] Nakamura M.T., Yudell B.E., Loor J.J. (2014). Regulation of energy metabolism by long-chain fatty acids. Prog. Lipid Res..

[9] Takeuchi Y., Yahagi N., Izumida Y., Nishi M., Kubota M., Teraoka Y., Yamamoto T., Matsuzaka T., Nakagawa Y., Sekiya M. (2010). Polyunsaturated fatty acids selectively suppress sterol regulatory element-binding protein-1 through proteolytic processing and autoloop regulatory circuit. J. Biol. Chem..

[10] Liput K.P., Lepczyński A., Ogłuszka M., Nawrocka A., Poławska E., Grzesiak A., Ślaska B., Pareek C.S., Czarnik U., Pierzchała M. (2021). Effects of Dietary n-3 and n-6 Polyunsaturated Fatty Acids in Inflammation and Cancerogenesis. Int. J. Mol. Sci..

[11] Minihane A.M. (2009). Nutrient gene interactions in lipid metabolism. Curr. Opin. Clin. Nutr. Metab. Care.

[12] Siculella L., Sabetta S., Damiano F., Giudetti A.M., Gnoni G.V. (2004). Different dietary fatty acids have dissimilar effects on activity and gene expression of mitochondrial tricarboxylate carrier in rat liver. FEBS Lett..

[13] Damiano F., Gnoni G.V., Siculella L. (2009). Functional analysis of rat liver citrate carrier promoter: Differential responsiveness to polyunsaturated fatty acids. Biochem. J..

[14] Salati L.M., Szeszel-Fedorowicz W., Tao H., Gibson M.A., Amir-Ahmady B., Stabile L.P., Hodge D.L. (2004). Nutritional regulation of mRNA processing. J. Nutr..

[15] Jump D.B. (2011). Fatty acid regulation of hepatic lipid metabolism. Curr. Opin. Clin. Nutr. Metab. Care.

[16] Duplus E., Glorian M., Forest C. (2000). Fatty acid regulation of gene transcription. J. Biol. Chem..

[17] Worsch S., Heikenwalder M., Hauner H., Bader B.L. (2018). Dietary n-3 long-chain polyunsaturated fatty acids upregulate energy dissipating metabolic pathways conveying anti-obesogenic effects in mice. Nutr. Metab..

[18] El-Gharba A., Vockley J. (2018). Inborn Errors of Metabolism with Myopathy: Defects of Fatty Acid Oxidation and the Carnitine Shuttle System. Pediatr. Clin. N. Am..

[19] Rubio-Gozalbo M.E., Bakker J.A., Waterham H.R., Wanders R.J. (2004). Carnitine-acylcarnitine translocase deficiency, clinical, biochemical and genetic aspects. Mol. Asp. Med..

[20] Longo N., Frigeni M., Pasquali M. (2016). Carnitine transport and fatty acid oxidation. Biochim. Biophys. Acta.

[21] Pierre G., Macdonald A., Gray G., Hendriksz C., Preece M.A., Chakrapani A. (2007). Prospective treatment in carnitine-acylcarnitine translocase deficiency. J. Inherit. Metab. Dis..

[22] Priore P., Stanca E., Gnoni G.V., Siculella L. (2012). Dietary fat types differently modulate the activity and expression of mitochondrial carnitine/acylcarnitine translocase in rat liver. Biochim. Biophys. Acta.

[23] Goto T. (2019). A review of the studies on food-derived factors which regulate energy metabolism via the modulation of lipid-sensing nuclear receptors. Biosci. Biotechnol. Biochem..

[24] Deckelbaum R.J., Torrejon C. (2012). The omega-3 fatty acid nutritional landscape: Health benefits and sources. J. Nutr..

[25] Gutgesell A., Wen G., König B., Koch A., Spielmann J., Stangl G.I., Eder K., Ringseis R. (2009). Mouse carnitine-acylcarnitine translocase (CACT) is transcriptionally regulated by PPARalpha and PPARdelta in liver cells. Biochim. Biophys. Acta.

[26] Mangiullo R., Gnoni A., Damiano F., Siculella L., Zanotti F., Papa S., Gnoni G.V. (2010). 3,5-diiodo-L-thyronine upregulates rat-liver mitochondrial F(o)F(1)-ATP synthase by GA-binding protein/nuclear respiratory factor-2. Biochim. Biophys. Acta.

[27] Rossmeisl M., Medrikova D., van Schothorst E.M., Pavlisova J., Kuda O., Hensler M., Bardova K., Flachs P., Stankova B., Vecka M. (2014). Omega-3 phospholipids from fish suppress hepatic steatosis by integrated inhibition of biosynthetic pathways in dietary obese mice. Biochim. Biophys. Acta.

[28] Kroupova P., van Schothorst E.M., Keijer J., Bunschoten A., Vodicka M., Irodenko I., Oseeva M., Zacek P., Kopecky J., Rossmeisl M. (2020). Omega-3 phospholipids from krill oil enhance intestinal fatty acid oxidation more effectively than omega-3 triacylglycerols in high-fat diet-fed obese mice. Nutrients.

[29] Shibabaw T. (2021). Omega-3 polyunsaturated fatty acids: Anti-inflammatory and anti-hypertriglyceridemia mechanisms in cardiovascular disease. Mol. Cell. Biochem..

[30] Katsnelson G., Ceddia R.B. (2020). Docosahexaenoic and eicosapentaenoic fatty acids differentially regulate glucose and fatty acid metabolism in L6 rat skeletal muscle cells. Am. J. Physiol. Cell. Physiol..

[31] Clarke S.D., Gasperikova D., Nelson C., Lapillonne A., Heird W.C. (2002). Fatty acid regulation of gene expression: A genomic explanation for the benefits of the mediterranean diet. Ann. N. Y. Acad. Sci..

[32] Lee J., Choi Y.R., Kim M., Park J.M., Kang M., Oh J., Lee C.J., Park S., Kang S.M., Manabe I. (2021). Common and differential effects of docosahexaenoic acid and eicosapentaenoic acid on helper T-cell responses and associated pathways. BMB Rep..

[33] Dias B.V., Gomes S.V., Castro M.L.D.C., Carvalho L.C.F., Breguez G.S., de Souza D.M.S., Ramos C.O., Sant’Ana M.R., Nakandakari S.C.B.R., Araujo C.M. (2022). EPA/DHA and linseed oil have different effects on liver and adipose tissue in rats fed with a high-fat diet. Prostaglandins Other Lipid Mediat..

[34] Emami K.H., Burke T.W., Smale S.T. (1998). Sp1 activation of a TATA-less promoter requires a species-specific interaction involving transcription factor IID. Nucleic Acids Res..

[35] Tonazzi A., Giangregorio N., Console L., Palmieri F., Indiveri C. (2021). The Mitochondrial Carnitine Acyl-carnitine Carrier (SLC25A20): Molecular Mechanisms of Transport, Role in Redox Sensing and Interaction with Drugs. Biomolecules.

[36] Vitoria I., Martín-Hernández E., Peña-Quintana L., Bueno M., Quijada-Fraile P., Dalmau J., Molina-Marrero S., Pérez B., Merinero B. (2015). Carnitine-acylcarnitine translocase deficiency: Experience with four cases in Spain and review of the literature. JIMD Rep..

[37] Iacobazzi V., Convertini P., Infantino V., Scarcia P., Todisco S., Palmieri F. (2009). Statins, fibrates and retinoic acid upregulate mitochondrial acylcarnitine carrier gene expression. Biochem. Biophys. Res. Commun..

[38] Tachibana K., Takeuchi K., Inada H., Yamasaki D., Ishimoto K., Tanaka T., Hamakubo T., Sakai J., Kodama T., Doi T. (2009). Regulation of the human SLC25A20 expression by peroxisome proliferator-activated receptor alpha in human hepatoblastoma cells. Biochem. Biophys. Res. Commun..

[39] Gacias M., Pérez-Martí A., Pujol-Vidal M., Marrero P.F., Haro D., Relat J. (2012). PGC-1β regulates mouse carnitine-acylcarnitine translocase through estrogen-related receptor α. Biochem. Biophys. Res. Commun..

[40] Convertini P., Infantino V., Bisaccia F., Palmieri F., Iacobazzi V. (2011). Role of FOXA and Sp1 in mitochondrial acylcarnitine carrier gene expression in different cell lines. Biochem. Biophys. Res. Commun..

[41] Louet J.F., Chatelain F., Decaux J.F., Park E.A., Kohl C., Pineau T., Girard J., Pegorier J.P. (2001). Long-chain fatty acids regulate liver carnitine palmitoyltransferase I gene (L-CPT I) expression through a peroxisome-proliferator-activated receptor alpha (PPARalpha)-independent pathway. Biochem. J..

[42] Bruni F., Polosa P.L., Gadaleta M.N., Cantatore P., Roberti M. (2010). Nuclear respiratory factor 2 induces the expression of many but not all human proteins acting in mitochondrial DNA transcription and replication. J. Biol. Chem..

[43] Yang Z.F., Drumea K., Mott S., Wang J., Rosmarin A.G. (2014). GABP transcription factor (nuclear respiratory factor 2) is required for mitochondrial biogenesis. Mol. Cell. Biol..

[44] Scarpulla R.C. (2002). Nuclear activators and coactivators in mammalian mitochondrial biogenesis. Biochim. Biophys. Acta.

[45] Kim H.J., Lee S.H., Jeong C., Han Y.H., Lee M.O. (2024). RORα-GABP-TFAM axis alleviates myosteatosis with fatty atrophy through reinforcement of mitochondrial capacity. J. Cachexia Sarcopenia Muscle.

[46] Ewaschuk J.B., Almasud A., Mazurak V.C. (2014). Role of n-3 fatty acids in muscle loss and myosteatosis. Appl. Physiol. Nutr. Metab..

[47] Ebadi M., Tsien C., Bhanji R.A., Dunichand-Hoedl A.R., Rider E., Motamedrad M., Mazurak V.C., Baracos V., Montano-Loza A.J. (2022). Myosteatosis in Cirrhosis: A Review of Diagnosis, Pathophysiological Mechanisms and Potential Interventions. Cells.

[48] Niopek K., Üstünel B.E., Seitz S., Sakurai M., Zota A., Mattijssen F., Wang X., Sijmonsma T., Feuchter Y., Gail A.M. (2017). A Hepatic GAbp-AMPK Axis Links Inflammatory Signaling to Systemic Vascular Damage. Cell. Rep..

[49] Nammo T., Udagawa H., Funahashi N., Kawaguchi M., Uebanso T., Hiramoto M., Nishimura W., Yasuda K. (2018). Genome-wide profiling of histone H3K27 acetylation featured fatty acid signalling in pancreatic beta cells in diet-induced obesity in mice. Diabetologia.

